# SARC-F and the Risk of Falling in Middle-Aged and Older Community-Dwelling Postmenopausal Women

**DOI:** 10.3390/ijerph182111570

**Published:** 2021-11-04

**Authors:** María Alzar-Teruel, Fidel Hita-Contreras, Antonio Martínez-Amat, María Leyre Lavilla-Lerma, Raquel Fábrega-Cuadros, José Daniel Jiménez-García, Agustín Aibar-Almazán

**Affiliations:** Department of Health Sciences, Faculty of Health Sciences, University of Jaén, 23071 Jaén, Spain; mariaalzarteruel@gmail.com (M.A.-T.); fhita@ujaen.es (F.H.-C.); amamat@ujaen.es (A.M.-A.); rfabrega@ujaen.es (R.F.-C.); josedanieljimenezgarcia@gmail.com (J.D.J.-G.); aaibar@ujaen.es (A.A.-A.)

**Keywords:** sarcopenia, fear of falling, balance confidence, anxiety, depression

## Abstract

(1) Background: The objective of the present study was to determine the ability of the SARC-F questionnaire to identify individuals at risk of falling among middle-aged and older community-dwelling postmenopausal women. (2) Methods: An analytical cross-sectional study was conducted on 157 women (70.80 ± 8.37 years). The SARC-F questionnaire was used to screen for risk of sarcopenia. Fear of falling and balance confidence, as measured by the Falls Efficacy Scale-International (FES-I) and the Activities-Specific balance Scale-16 items (ABC-16) respectively, were used to assess risk of falling. Anxiety and depression (Hospital Anxiety and Depression Scale), fatigue (Fatigue Severity Scale), body mass index, waist-to-hip ratio, and sleep duration were also determined. (3) Results: Logistic regression showed that higher risk of falling as assessed by FES-I was associated with higher SARC-F scores (OR = 1.656), anxiety levels (OR = 1.147), and age (OR = 1.060), while increased SARC-F scores (OR = 1.612), fatigue (OR = 1.044), and shorter sleep duration (OR = 0.75) were related to ABC-16 scores. In addition, a SARC-F cutoff of 1.50 (83.33% sensitivity and 59.13% specificity) and 3.50 (44.44% sensitivity and 89.26% specificity) were shown to be able to discriminate participants at risk of falling according to the FES-I and the ABC-16, respectively. (4) Conclusions: our results show that SARC-F is an independent predictor of the risk of falling among middle-aged and older community-dwelling postmenopausal women.

## 1. Introduction

Falls are a serious clinical problem among community-dwelling older adults, with almost one third of those aged 65 years and over experiencing at least one fall each year [1]. For the last thirty years, disability and mortality related to falls among older people have increased, and consequently fall prevention is critical [2]. It has been reported that, compared with men, women have a higher rate of nonfatal falls and fall-related injuries for all age groups [3,4]. Sayyah et al. [5] described the probability of falls for women over 65 years being 31.9%. This, together with the high prevalence of osteoporosis in older women due to menopause, highlights the importance of preventing falls among postmenopausal women.

Due to the complex nature of falls, several fall-risk factors have been described. These are usually classified as either extrinsic (i.e., home hazards, footwear, or medication) and intrinsic factors, which can be modifiable or not modifiable, and include both physical and psychological factors [6]. Balance confidence and fear of falling are two of the most important psychological factors related to balance impairment and falling in older adults [7]. Fear of falling refers to a lasting concern about falling that can ultimately lead to an avoidance of activities of daily life [8], and balance confidence relates to the individual’s confidence in their abilities to maintain balance and not fall when performing daily life activities [9]. Additionally, the transition to the menopause status is associated with changes in body composition, and it has been shown that a BMI of ≤25 kg/m^2^ and android body-fat distribution are independent fall risk factors [10]. Moreover, menopause has been described as an independent predictor of decreased muscle strength and balance, two important risk factors of falling [11].

Sarcopenia is a major clinical problem for older people, and is defined by low levels of muscle strength and mass, with poor physical performance as an indicator of severity [12]. Sarcopenia is associated with a higher risk of health-related adverse events, such as falls, fractures, functional decline, higher incidence of hospitalizations, and mortality [13]. The Strength, Assistance with Walking, Rise from a Chair, Climb Stairs, Falls (SARC-F) is a simple and inexpensive questionnaire developed to screen for persons with sarcopenia [14]. The second meeting of the European Working Group on Sarcopenia in Older People (EWGSOP2), that took place in 2018, recommended the use of the SARC-F in clinical practice as an inexpensive and convenient way to identify individuals at risk of sarcopenia [12]. Sarcopenia has been positively associated with falls and fractures in older adults [15,16], and a recent meta-analysis concluded that this association could be found in community-dwelling older adults, but not among older people in nursing homes [17].

The objective of the present study was to determine, taking into account some possible confounders (anxiety, depression, fatigue sleep duration severity, age, anthropometric measures, history of falls, and osteoporosis), the ability of the SARC-F questionnaire to identify people at risk of falling among middle-aged and older community-dwelling postmenopausal women. We hypothesized that the SARC-F questionnaire, after adjusting for possible confounders, is an independent predictive factor of fall risk, regarding balance confidence and the fear of falling in middle-aged and older community-dwelling postmenopausal women.

## 2. Materials and Methods

### 2.1. Study Design and Participants

**Figure 1:**
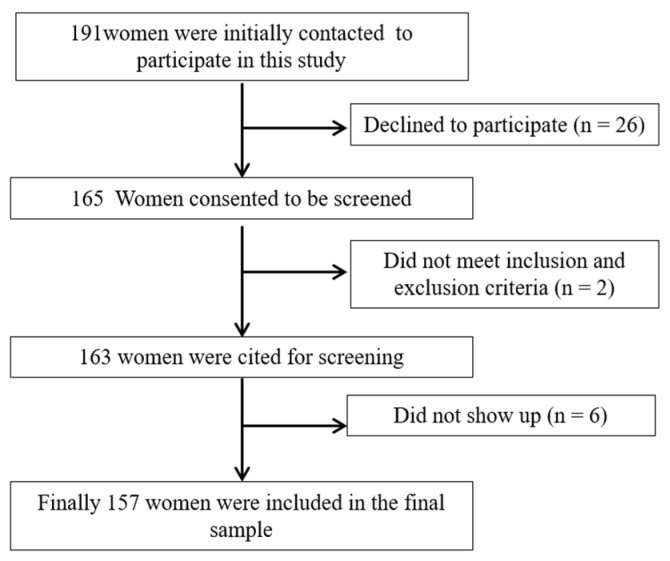
Flowchart diagram of the participants in this study.

This study was conducted following the Declaration of Helsinki, good clinical practices, and all applicable laws and regulations. A written informed consent to participate in this study was signed and provided by the participants. Inclusion criteria were ambulant women aged 50 years or older, with at least twelve months of amenorrhea, and able to understand the instructions, programs, and protocols involved in the study. Exclusion criteria: conditions or disorders that could affect balance and functional activity (such as auditory or vestibular alterations), central or peripheral neurological diseases, serious psychiatric or somatic diseases that might influence their responses to the questionnaires and tests, and taking sedatives or sleep medication. An analytical, cross-sectional study was conducted on 157 women out of the 191 who were initially contacted. This study was carried out from February 2020 to November 2020. Participants were recruited via e-mail and telephone after contacting four associations for older adults in Jaén (Spain). Figure 1 shows a flowchart diagram of the participants. This research was approved by the Research Ethics Committee of Jaén, Spain (OCT.18/4.PRY).

### 2.2. Outcomes

The following self-reported data were collected face-to-face by well-trained researchers: age (years), years since the onset of menopause (menopause being defined as not having the menstrual period within the last 12 months), occupational status (retired, working, or unemployed), marital status (single, married/cohabiting, or separated/divorced/widowed), educational level (no formal education, primary, secondary, or university education), the presence of osteoporosis (clinically diagnosed), and sleep duration (through the question “how many hours of sleep do you usually get in a 24 h period?”). Their history of falls was addressed by the question “have you experienced a fall in the last twelve months?”. A fall was defined as “an unexpected event in which the participant came to rest on the ground, floor, or lower level” [18].

As for anthropometric parameters, we measured height (m) and weight (kg) with an adult height scale (T201-T4 Asimed) and a 100 g–130 kg precision digital weight scale (Tefal), respectively. In order to calculate body mass index (BMI), weight was divided by height squared. BMI values under 25 reflect normal weight, from 25 to 29.99 indicate overweight, and values of 30 and over identify obesity [19]. A 1.5 m flexible tape was used to measure waist and hip circumferences (cm), and waist-to-hip ratio (WHR) was calculated by dividing waist by hip circumference. WHR values under 0.76 and over 0.86 reflect gynoid and android body fat distribution patterns, respectively, while values ranging from 0.76 to 0.85 indicate a uniform body fat distribution pattern [20].

#### 2.2.1. Risk of Sarcopenia

The risk of sarcopenia was assessed by the SARC-F questionnaire [14,21], which evaluates the limitations experienced concerning five items or components: strength, walking assistance, rising from a chair, climbing stairs, and the number of falls in the previous year. This questionnaire provides a total score ranging from 0 to 10, with greater scores reflecting increased risk of sarcopenia. Values ≥ 4 are a sign that individuals are at risk of sarcopenia.

#### 2.2.2. Fear of Falling

Fear of falling values were calculated using the Falls Efficacy Scale-International (FES-I) [22,23]. This questionnaire comprises 16 items that assess a variety of physical, social, and functional aspects related with the fear of falling. The FES-I score ranges from 16 (complete absence of fear of falling) to 64 (extreme concern). It has been described that a score over 26 points can predict the future occurrence of falls in women aged ≥50 years [24].

#### 2.2.3. Balance Confidence

In order to assess the level of balance confidence when performing activities of daily living [9,25], we used the Activities-specific Balance Confidence scale, 16 items (ABC-16). It is comprised of 16 items (0–100%) that are summarized into a total score obtained by the addition of all the items (0–1600) divided by 16. Higher scores (percentage) represent greater balance confidence. It has been shown that a score over 67% is a reliable cutoff for predicting the occurrence of a future fall in older adults [26].

#### 2.2.4. Anxiety and Depression

The level of anxiety and depression symptoms were assessed by the Hospital Anxiety and Depression Scale (HADS) [27,28]. This instrument consists of 14 items distributed into two subscales of seven items each (with scores ranging from 0 to 21), one for anxiety and another for depression. Higher scores indicate greater severity of the symptoms. Values over 11 denote clinically relevant symptoms.

#### 2.2.5. Fatigue Severity

The Fatigue Severity Scale was used to assess self-perceived fatigue severity during the last seven days. This scale comprises nine items (with scores ranging from one to seven) that are summed to obtain a total score (ranging 9–63), with a greater score indicating greater self-perceived fatigue. A score ≥ 36 indicates severe fatigue [29].

### 2.3. Sample Size Calculation

Following the recommendations of Ortega Calvo and Cayuela Domínguez [30], at least ten participants per variable were required in the logistic regression model. Given that we employed ten possible predicting variables linked to sleep quality (SARC-F, age, BMI, WHR, anxiety, depression, sleep duration, self-reported fatigue, history of falls, and the presence of osteoporosis), over 100 subjects should be included in the study. The final number of participants was 157.

### 2.4. Statistical Analysis

Continuous and categorical variables were presented as means and standard deviations, and as frequencies and percentages, respectively. The chi-square and Student’s t tests were employed to analyze the possible individual differences in SARC-F regarding risk of falling as assessed by the FES-I and the ABC questionnaires, as well as other possible confounders, such as age, BMI, WHR, anxiety and depression, sleep duration, self-reported fatigue, history of falls, and the presence of osteoporosis (independent variables). Variables showing significant individual differences (*p* < 0.05) were chosen for the stepwise logistic regression model. The odds ratio (OR) is deemed as significant when the 1.00 value is not included in the 95% confidence interval (CI). In order to evaluate the overall goodness-of-fit of the model, we used the chi-square and Hosmer–Lemeshow tests, as well as the Cox and Snell R^2^ and Nagelkerke R^2^. The accuracy of the SARC-F score in discriminating between women at risk of falling (assessed with the FES-I and the ABC-16 cutoffs) was evaluated using receiver operating characteristic (ROC) curve analysis. In a ROC curve, the true-positive rate (sensitivity) is calculated in accordance with the false-positive rate for different cutoff points. Each of the points described in the ROC curve represents a sensitivity/specificity pair corresponding to a particular decision threshold [31]. The area under the ROC curve (AUC) was also calculated as a measure of how well a parameter is able to distinguish between two diagnostic groups (with and without risk of falling). AUC was considered statistically significant when the 0.5 value was not included in the 95% confidence interval (CI). A 95% confidence level was used (*p* < 0.05). Data management and analysis were carried out using the SPSS statistical package for the social sciences for Windows (SPSS Inc., Chicago, IL, USA).

## 3. Results

Baseline descriptive characteristics of the 157 participants in this study (70.80 ± 8.37 years) are presented in Table 1. Most of them were retired (62.42%) and had primary education or less (64.97%), 35.67% had experienced at least one fall in the last 12 months, and 37.58% had osteoporosis. As for the anthropometric data, BMI values pointed to overweight and WHR to uniform body fat distribution (although in the limit of android pattern). Anxiety, depression sleep duration, and fatigue were in the normal ranges. The SARC-F mean score indicated low risk of sarcopenia, and those of the ABC-16 and FES-I suggested a low risk of falling.

Table 2 displays the individual differences between the SARC-F questionnaire and the confounding variables analyzed in this study with respect to the risk of falling, according to the FES-I cutoff. Participants who were at risk of falling had significantly greater SARC-F scores (*p* < 0.001), age (*p* < 0.001), as well as higher levels of self-perceived fatigue (*p* < 0.001), anxiety (*p* < 0.001), and depression (*p* < 0.001) symptoms. Women with osteoporosis (*p* = 0.021) were at high risk of experiencing a fall.

The analysis of the individual differences between the SARC-F questionnaire and other possible confounders according to the ABC-16 cutoff for risk of falling is presented in Table 3. Just as with FES-I, individuals with greater SARC-F scores (*p* < 0.001) and self-perceived fatigue (*p* < 0.001), anxiety (*p* = 0.023), and depression (*p* = 0.032) symptoms were at risk of falling according to the ABC-16, as well as those with decreased sleep duration (*p* = 0.0319) and a history of falls (*p* = 0.041).

In order to analyze if SARC-F scores and other variables were independently associated with fall risk according to the FES-I and the ABC-16 scores, a logistic regression was performed (Table 4). Regarding the FES-I score, SARC-F score (*p* = 0.002), anxiety (*p* = 0.012), and age (*p* = 0.031) were independent factors for increased risk of falling as evaluated by the FES-I. The adequacy of the multivariate logistic regression model was proved by the Hosmer–Lemeshow goodness of fit test (chi-square = 7.055, *p* = 0.531). The model explained 22.31 (Cox and Snell R^2^) and 32.48 (Nagelkerke R^2^) of the variance in FES-I scores, and correctly classified 80.25% of all cases. As for the risk of falling assessed by the ABC cutoff score, SARC-F score (*p* = 0.001), self-perceived fatigue (*p* = 0.001), and shorter sleep duration (*p* = 0.053) remained independent predictive factors. The Hosmer–Lemeshow goodness of fit test demonstrated the adequacy of the multivariate logistic regression model (chi-square = 10.735, *p* = 0.217). The model as a whole explained between 17.61 and 26.71 (Cox and Snell R^2^ and Nagelkerke R^2^, respectively) of the ABC-16 variance, and correctly classified 80.89% of all cases.

**Figure 2:**
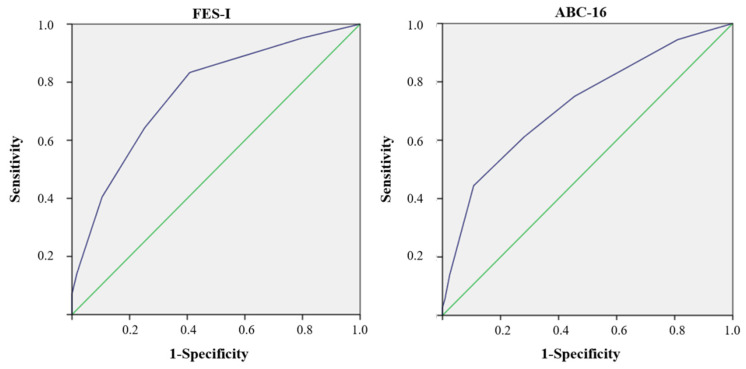
The ROC curves of FES-I and ABC-16. 1-Specificity: false-positive rate. ABC-16: Activities-Specific Balance Confidence scale-16 items. FES-I: Falls-Efficacy Scale-International. ROC: receiver-operating characteristic. The blue line represents the ROC curve and the green line is the reference line.

Finally, in order to study the accuracy of the SARC-F in discriminating women at risk of falling according to the FES-I and ABC-16, ROC curves were performed (Figure 2). The AUC for FES-I was 0.763 (95% IC 0.678–0.848), with a cutoff point of 1.50, 83.33% sensitivity, and 59.13% specificity, while the AUC for the ABC-16 was 0.722 (95% IC 0.623–0.820), with a cutoff point of 3.50, 44.44% sensitivity, and 89.26% specificity.

## 4. Discussion

The objective of this study was to evaluate the possible associations between SARC-F scores and the risk of falling, as assessed through balance confidence and fear of falling measurements in community-dwelling middle-aged and older postmenopausal women. Our results showed that, taking into account possible confounders, such as age, BMI, WHR, anxiety, depression, sleep duration, self-reported fatigue, history of falls, and osteoporosis, the SARC-F is an independent predictor of the risk of falling in this population.

Yang et al. [32] recorded that the recent modification of the definition of sarcopenia, carried out by the EWGSOP2 in 2018, is better suited to predict the incidence of the number of falls than the previous definition proposed by the EWGSOP1 [33]. In our results, the mean score obtained by the participants in the SARC-F questionnaire was 1.98, which denotes low risk of sarcopenia, with 26.75% of the participants being at risk for this condition. This is comparable to the data reported by Marincolo et al. [34], and higher than the 20.5% described by Lee et al. [35] for community-dwelling older adults. The difference with the latter could be explained by the lower mean BMI (24.16 kg/m^2^) and age (67.9 years) of participants, since obesity and older age are two important factors associated with sarcopenia [6].

Fear of falling has a multifactorial origin. Some research has documented the influence of gender on this psychological phenomenon, which occurs more frequently in women. In addition, a greater fear of falls and a greater number of falls have also been recorded in people with impaired functional and cognitive capacity [36,37]. On the other hand, other published articles have provided evidence about the relation between a greater fear of falling and an increase in the frequency of falls and fall-related comorbidities [38]. Our results showed that an increased risk of sarcopenia (as assessed with the SARC-F questionnaire) was an independent predictor of the risk of falling (as assessed with the FES-I). Moreover, we found that a score of 1.50 in the SARC-F score was able to discriminate people at risk of falling according to the FES-I, with a sensitivity of 83.33% and a specificity of 59.13%. Our logistic regression model also showed that older age and higher levels of anxiety were independently associated with a FES-I > 26. Our findings are in line with previous results. Cao et al. [39] found in a pilot study performed on older women, that participants at a high risk of sarcopenia (SARC-F ≥ 4) reported increased fear of falling, as measured by FES-I. In addition, Gadelha et al. [40] reported that higher FES-I scores were associated with all sarcopenia stages according to the EWGSOP criteria (first meeting) [33] among community-dwelling older women. However, Bahat Öztürk et al. [41], in a study conducted on 1021 community-dwelling adults aged ≥ 60 years, observed that the fear of falling was independently related to anxiety, as well as to limitations in activities of daily living and being of the female gender, but there were no associations with neither SARC-F nor handgrip strength. This difference could be explained by the fact that participants in the latter study scored considerably lower in SARC-F (median value of 1).

As for the association between sarcopenia and falls, Marincolo et al. [34] described a higher prevalence of falls in older adults with probable sarcopenia according to the EWGSOP2 and SARC-F (≥ 4 points). A recent systematic review and meta-analysis concluded that sarcopenic older adults were at a significantly higher risk of falls and fractures compared with non-sarcopenic equivalents, independently of the sarcopenia definition and other confounders [15]. It has been reported that balance confidence (ABC-16) was the best predictor of falling among older adults, followed by the fear of falling (FES-I) [7]. To the best of our knowledge, few studies have analyzed the associations between sarcopenia and balance confidence. Kirk et al. [42] have recently described that sarcopenic older persons had poorer balance confidence and a greater fear of falling. Nevertheless, a previous study showed that sarcopenic obesity, but not any of the sarcopenia stages, was linked to fall risk according to the FES-I [43], although EWGSOP1 criteria [33] were used in that work. The results of the present study suggested that a SARC-F score of 4 points and over was an independent predictor of fall risk as assessed by the ABC-16, but there was no association with BMI. In addition, we found that a SARC-F score of 3.50 could identify individuals at risk of falling (ABC-16), with a sensitivity of 44.44% and a specificity of 89.26%.

Finally, as for the confounding variables considered in this study, older age, depression, and anxiety, as well as being female, have been described as risk factors associated with a greater fear of falling and poor balance confidence among older people [44,45,46]. In the present study, besides SARC-F, anxiety and age remained independent fall risk predictors according to the FES-I, but not the ABC-16 cutoffs. Rivasi et al., in a study with a sample of community-dwelling people aged ≥60 years, found that, among others, participants who were older at baseline and had higher levels of depression and anxiety developed a fear of falling in a two-year follow up, although only the first two factors remained in the multivariate analysis [47]. Finally, sleep problems and greater levels of self-perceived fatigue have been associated with decreased balance confidence and greater fall risk in community-dwelling older adults [48,49]. This is in accordance with our findings, in which a high risk of falling assessed by the ABC-16 was related to anxiety and shorter sleep duration, as well as with SARC-F scores.

This study has some limitations that should be noted. Due to its cross-sectional design, it was not possible to determine fall incidence or causality. Information regarding chronic diseases and medications, fitness status, and the lifestyle of participants were not collected. In addition, this research was performed in middle-aged and older postmenopausal women from a particular geographical region, and any generalization of its results should be limited to individuals of similar characteristics. Future research should consider conducting prospective designs, recording the number of falls, chronic diseases, medications, and physical activity level, as well as including a more diverse population.

## 5. Conclusions

The results of this study suggest that SARC-F is an independent predictor of the risk of falling in middle-aged and older community-dwelling postmenopausal women. More specifically, increased SARC-F score, older age, and greater anxiety symptoms were associated with increased risk of falling as determined by the FES-I. On the other hand, higher SARC-F scores and self-perceived fatigue, as well as shorter sleep duration, were linked to an elevated risk of falling according to ABC-16. Furthermore, SARC-F cutoffs of 1.50 (83.33% sensitivity and 59.13% specificity) and 3.50 (44.44% sensitivity and 89.26% specificity) could discriminate participants at a risk of falling, according to the FES-I and the ABC-16, respectively.

## Figures and Tables

**Table 1:** Descriptive data of the participants in this study.

Study Sample = 157			
		Mean	SD
Age (Years)		70.80	8.37
Years in menopause		21.41	8.91
BMI		29.28	4.33
WHR		0.86	0.09
SARC-F		1.98	1.53
HADS- anxiety		6.05	4.11
HADS-depression		4.90	3.50
Sleep duration (hours)		6.57	1.45
Self-perceived fatigue		22.19	15.28
FES-I		23.54	7.34
ABC-16		76.28	17.71
		Frequency	Percentage
Occupational status	Retired	98	62.42
	Working	30	19.11
	Unemployed	29	18.47
Marital status	Single	6	3.82
	Married/cohabiting	76	48.41
	Separated/divorced/ Widowed	75	47.77
Educational level	No formal education	27	17.20
	Primary	75	47.77
	Secondary	41	26.11
	University	14	8.92
History of falls	No	101	64.33
	Yes	56	35.67
Osteoporosis	No	98	62.42
	Yes	59	37.58

ABC-16: Activities-Specific Balance Confidence scale-16 items. BMI: body mass index. FES-I: Falls-efficacy Scale-International. HADS: Hospital Anxiety and Depression Scale. SARC-F: Strength, Assistance in Walking, Rise from a Chair, Climb Stairs, and Falls. SD: standard deviation. SMI: skeletal muscle mass index. WHR: waist-to-hip ratio.

**Table 2:** Individual differences in SARC-F and possible confounders regarding the risk of falling (FES-I).

		Risk of Falling (FES-I)				
		No (n = 115)		Yes (n = 42)		
		Mean	SD	Mean	SD	*p*-Value
SARC-F		1.58	1.30	3.07	1.58	<0.001
Age		69.39	7.71	74.64	8.96	<0.001
BMI		29.09	4.01	29.83	5.10	0.342
WHR		0.85	0.10	0.87	0.06	0.262
Self-perceived fatigue		19.66	14.94	29.12	14.17	<0.001
Anxiety		5.30	4.08	8.10	3.48	<0.001
Depression		4.23	3.25	6.74	3.54	<0.001
Sleep Duration		6.70	1.44	6.19	1.45	0.050
		Frequency	Percentage	Frequency	Percentage	
History of falls	No (n = 101)	79	68.70%	22	520.38%	0.059
	Yes (n = 56)	36	31.30%	20	470.62%	
Osteoporosis	No (n = 98)	78	67.8%	20	470.6%	0.021
	Yes (n = 59)	37	32.2%	22	520.4%	

BMI: body mass index. FES-I: Falls-Efficacy Scale-International. SARC-F: Strength, Assistance in Walking, Rise from a Chair, Climb Stairs, and Falls. SD: standard deviation. WHR: waist-to-hip ratio.

**Table 3:** Individual differences in SARC-F and possible confounders regarding the risk of falling (ABC-16).

		Risk of Falling (ABC-16)				
		No (n = 115)		Yes (n = 42)		
		Mean	SD	Mean	SD	*p*-Value
SARC-F		1.69	1.35	2.97	1.68	<0.001
Age		70.31	8.14	72.44	9.01	0.179
BMI		29.09	4.01	29.94	5.28	0.302
WHR		0.86	0.10	0.87	0.07	0.445
Self-perceived fatigue		19.23	13.82	32.14	15.93	<0.001
Anxiety		5.64	4.11	7.42	3.82	0.023
Depression		4.58	3.32	6.00	3.89	0.032
Sleep Duration		6.71	1.40	6.07	1.53	0.019
		Frequency	Percentage	Frequency	Percentage	
History of falls	No (n = 101)	83	68.60	18	50.00	0.041
	Yes (n = 56)	38	31.40	18	50.00	
Osteoporosis	No (n = 98)	78	64.46	20	55.56	0.333
	Yes (n = 59)	43	35.54	16	44.44	

ABC-16: Activities-specific Balance Confidence scale-16 items. BMI: Body Mass Index. SARC-F: Strength, Assistance in walking, Rise from a chair, Climb stairs, and Falls. SD: Standard deviation. WHR: Waist to Hip Ratio.

**Table 4:** Multivariate logistic regression analyses for risk of falling regarding FES-I and ABC-16 scores.

		Exp (B)	95 CI		*p*-Value
			Inferior	Superior	
FES-I	SARC-F	1.656	1.212	2.263	0.002
	Anxiety	1.147	1.031	1.276	0.012
	Age	1.060	1.005	1.118	0.031
ABC-16	SARC-F	1.612	1.201	2.165	0.001
	Self-perceived fatigue	1.044	1.017	1.072	0.001
	Sleep duration	0.745	0.553	1.004	0.053

ABC-16: Activities-Specific Balance Confidence scale-16 items. CI: confidence interval. FES-I: Falls-Efficacy Scale-International. SARC-F: Strength, Assistance in Walking, Rise from a Chair, Climb Stairs, and Falls.

## Data Availability

The data shown in this study are available upon request from the corresponding author. The data is not available to the public, since taking into account the sensitive nature of all the questions asked in this study, all participants were guaranteed that the data obtained would be confidential and would not be shared.
